# Generating Mutant Renal Cell Lines Using CRISPR Technologies

**DOI:** 10.1007/978-1-4939-9841-8_20

**Published:** 2020-01-01

**Authors:** Nuria Perretta-Tejedor, Grace Freke, Marian Seda, David A. Long, Dagan Jenkins

**Keywords:** Gene editing, sgRNA, pX330, IMCD3, HEK293, Transfection, Fluorescence-activated cell sorting

## Abstract

Gene editing using the CRISPR/Cas9 system is an extremely efficient approach for generating mutations within the genomic DNA of immortalized cell lines. This procedure begins with a straightforward cloning step to generate a single plasmid encoding the Cas9 enzyme as well as a synthetic guide RNA (sgRNA) which is selected to target specific sites within the genome. This plasmid is transfected into cells either alone, in order to generate random insertion-deletion alleles (“indels”) at the desired locus via the nonhomologous end-joining pathway, or in conjunction with a homology-directed repair template oligonucleotide to generate a specific point mutation. Here we describe a procedure to perform gene editing in IMCD3 and HEK293 cells and to subsequently isolate clonal cell lines carrying mutations of interest.

## 1 Introduction

**Fig. 1:**
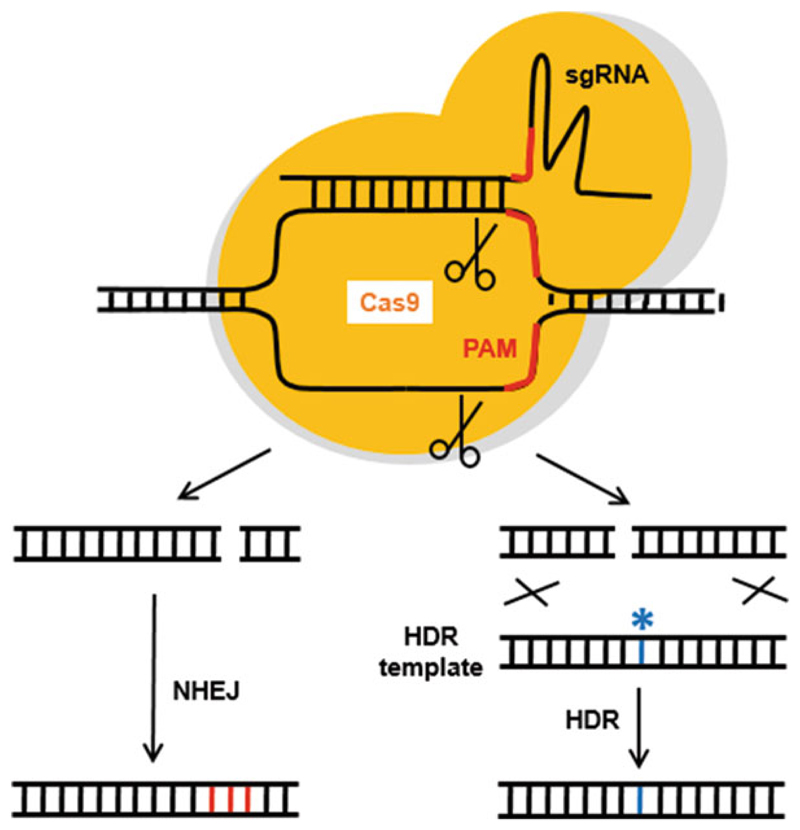
Gene editing using CRISPR-Cas9. Schematic showing the invasion of a sgRNA into a DNA double helix in concert with Cas9. The sgRNA consists of a short region of homology coupled to a PAM sequence as well as a more complex region with secondary structure. The region of homology is all that is cloned into plasmid pX330 in this protocol. Cas9 cuts the DNA at the PAM sequence causing a double-strand break. This can be repaired either by the NHEJ pathway or, in conjunction with a template oligo, by HDR. These pathways typically result in the introduction of indels (red) or point mutations (blue)

In recent years, there has been huge growth in the application of gene-editing technologies. The concept of gene editing began in the late 1990s with the development of zinc-finger nucleases (ZFNs) [1] and subsequently with transcription-like effector nucleases (TALENs) [2–4]. However, both of these technologies are limited by their relatively low targeting efficiencies, the relative rarity of suitable ZFN cut sites within the genome, and the need for complex cloning procedures to generate TALEN DNA-binding oligonucleotides. These limitations were circumvented by the development of the clustered regularly interspaced short palindromic repeats/CRISPR-associated endonuclease (CRISPR/ Cas9) system [5, 6], following the observation that the type II CRISPR system from *Streptococcus pyogenes* (SpCas) could be engineered to edit mammalian genomes [7, 8]. This editing system combines the Cas9 endonuclease with a synthetic guide RNA (sgRNA) that acts as a signal to deliver Cas9 to a specific genomic target sequence where it can induce a double-strand break (DSB; Fig. 1). Cas9 recognizes a protospacer adjacent motif (PAM) located immediately downstream of its sgRNA in order to elicit its function. DSBs can be repaired by one of the two mechanisms which act according to the presence or absence of a donor DNA template: nonhomologous end-joining (NHEJ) or homology-directed repair (HDR). NHEJ is error-prone, often introducing base insertions or deletions (indels), typically leading to a frameshift mutation. On the other hand, HDR employs a repair template with homology to the target site [9, 10]. To verify the efficiency of Cas9 cleavage at the target site we employ the T7 endonuclease (T7EI) assay. T7EI recognizes and cleaves DNA heteroduplexes containing mutant DNA sequences following PCR amplification of the targeted site and reannealing, and serves as a simple means of gauging targeting efficiency and screening for clonal cell lines that carry mutations [11–13].

Here we describe a procedure which we have adapted from the Zhang Lab at the Broad Institute [9], in order to generate frame-shift and point mutations in renal cell lines. Key features of this protocol include the following: (1) sorting of cells co-transfected with GFP together with the Cas9/sgRNA-expressing plasmid enhances the overall targeting efficiency. (2) We culture cells for at least 2 weeks following transfection of these plasmids before sorting single cells into clonal cell cultures, as we have found that shorter periods of time post-transfection are associated with a higher level of mosaic mutations, presumably owing to continued expression of Cas9/sgRNA. (3) For introduction of point mutations via HDR, we have found that a template oligo consisting of 60 nucleotides on either side of the DNA mismatch has good targeting efficiency (*see* **Note 1**).

To date, our lab has successfully employed these methods to produce immortalized renal cells carrying desired mutations (and defined genotypes) at over 60 genomic loci. We have predominantly used inner medullary collecting duct (IMDC3) cells, which are mouse epithelial cells allowing us to study the genes involved in cilia function, and human embryonic kidney (HEK293) cells, which have enabled us to model features of human renal diseases including polycystic kidney disease. We have also used this protocol successfully in other cell lines, including Chinese hamster ovary (CHO) cells, and the techniques could be used to edit genes in glomerular podocytes and endothelium, cells particularly affected in diabetic nephropathy. In other works, we have found that analysis of multiple clonal IMCD3 or HEK293 cell lines carrying biallelic mutations in genes of interest can have the advantage of reducing phenotypic variability, as compared to results generated using primary cell lines or transient gene-knockdown approaches, thereby revealing novel cellular phenotypes [14, 15]. The improved phenotypic consistency of these cellular models means that the CRISPR/Cas9 system is invaluable for studies of disease mechan-isms and for drug screening approaches.

## 2 Materials

### 2.1 Cloning of sgRNA into Plasmid pX330

1. Oligos 1 and 2 (designed in Subheading 3.1).
2. PCR primers (designed in Subheading 3.1).
3. pX330-U6-Chimeric_BB-CBh-hSpCas9 (Addgene plasmid ID: 42230).
4. BbsI enzyme (5000 U/mL; NEB).
5. 10× T4 ligase buffer (NEB).
6. T4 ligase enzyme (NEB).
7. *E. coli* JM109 competent cells (Promega).
8. Super Optimal broth with Catabolite repression (SOC) medium.
9. Luria Broth (LB) agar Amp plate: LB agar plate containing 100 μg/mL ampicillin.
10. LB Amp medium: LB medium containing 100 μg/mL ampicillin.
11. QIAprep Spin Miniprep kit (QIAGEN).
12. pX330 sequencing primer: 5′-CGTAACTTGAAAGTATTT CGATTTCTTGGC-3′.

### 2.2 Cell Culture and FACS

1. HEK293 cells (ATCC CRL-1573).
2. IMCD3 cells (ATCC CRL-2123).
3. Dulbecco PBS, no calcium, no magnesium (Gibco).
4. Medium for HEK293 cells: DMEM with GlutaMAX (Gibco) supplemented with 10% FBS (and 1% penicillin-streptomycin if desired).
5. Media for IMCD3 cells: DMEM/F-12 with GlutaMAX media (Gibco) supplemented with 10% FBS (and 1% penicillin- streptomycin if desired).
6. Trypsin for HEK293 cells: Trypsin-EDTA 0.05% (Gibco).
7. Trypsin for IMCD3 cells: Trypsin-EDTA 0.25% (Gibco).
8. FuGENE 6 (Promega).
9. Opti-MEM reduced-serum medium (Gibco).
10. pAcGFP1-C1 (Clontech).
11. Lipofectamine 2000 transfection reagent (Thermo Fisher).
12. MoFlo XDP (Beckman Coulter).

### 2.3 Genotyping Analysis

1. Molecular kit to extract genomic DNA (e.g., DNeasy Blood and Tissue Kit from Qiagen).
2. Pwo Master (Sigma-Aldrich).
3. 10× NEBuffer 2 (NEB).
4. T7 endonuclease I (NEB).
5. Agarose.
6. 1× TAE buffer (Invitrogen).
7. 1 kb DNA ladder (NEB).

## 3 Methods

**Fig. 2:**
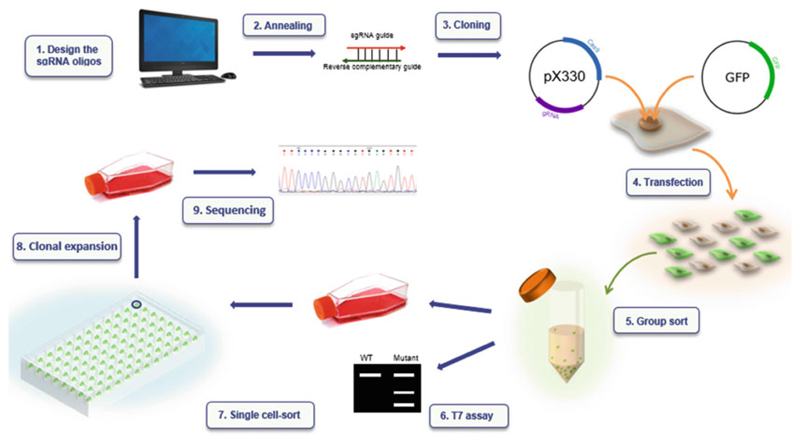
Overview of methodology. A sgRNA is designed and ordered as two oligos (1), which are annealed (2) and cloned into plasmid pX330 to yield plasmid pX330-sgRNA (3), which contains a Cas9 expression cassette and a sgRNA expression cassette. IMCD3 or HEK293 cells are transfected with pX330-sgRNA and pAcGFP1-C1, a GFP expression vector used as a positive marker of transfection (4). Fluorescence-activated cell sorting (FACS) is used for enrichment of GFP-positive cells in a step we term the “group sort” (5); these cells are presumed to have also successfully been transfected with pX330-sgRNA. Half of the group-sorted cells are reseeded and subcultured for 2 weeks (*see* **Note 15**). gDNA is extracted from the remaining cells and analyzed by T7 endonuclease I (T7EI) assay (6), to detect indels at the genomic target site within the cell population. After 2 weeks of subculture, the reseeded CRISPR cells are subjected to FACs again, and a single cell is seeded into each well of a 96-well plate, in a step that we call the “single-cell sort” (7) which is followed by clonal expansion (8). Each single cell has the propensity to establish a clonal colony, if it proliferates. Colonies are genotyped by PCR and Sanger sequencing to confirm the presence and nature of mutations in each (9)

The methods presented below should be used to generate random indels by NHEJ. A variation of this protocol can be used to generate a defined single base change mutation (or if required, substitution, insertion, or deletion of a few nucleotides) by HDR (*see* **Note 1**). For simplicity, the following methods describe the design and use of one sgRNA (*see* **Note 2**; Fig. 2).

### 3.1 sgRNA Design

1. Find your gene of interest within the relevant species on the Ensembl genome browser (www.ensembl.org). For HEK293 search the human genome, and for IMCD3 search the mouse genome.
2. Select a 23–500-nucleotide (nt) genomic region that you wish to edit (*see* **Note 3**).
3. Open the CRISPOR design tool (crispor.tefor.net). Select the appropriate target genome and paste your sequence into the search box. Submit the search. The online tool will produce a ranked list of all possible sgRNAs within the genomic sequence (*see* **Note 4**).
4. Identify a suitable sgRNA sequence from the displayed list (*see* **Note 5**).
5. Take the 20 nt sgRNA sequence (without the PAM). If its first nucleotide is not a “G,” then add a “G” at the 5′ end. This sequence will form the foundations of oligo 1.
6. Find the reverse complement of the sgRNA sequence (including the extra “G” nucleotide, if added). This will form the foundations of oligo 2.
7. To the 5′ end of oligo 1, insert nucleotides “CACC.” Design of oligo 1 is now complete.
8. To the 5′ end of oligo 2, insert nucleotides “AAAC.” Design of oligo 2 is now complete.
9. Order oligos 1 and 2 as 5′ phosphorylated oligos.
10. Design a pair of PCR primers flanking the sgRNA genomic target site (*see* **Note 6**).

### 3.2 Cloning of sgRNA into Plasmid pX330

#### 3.2.1 Annealing of Oligos 1 and 2

1. Resuspend each oligo (ordered in Subheading 3.1, **step 9**) to a concentration of 100 μM in Milli-Q water.
2. In a 0.2 mL PCR tube, combine 1 μL oligo 1 (100 μM), 1 μL oligo 2 (100 μM), and 8 μL Milli-Q water.
3. Anneal in a thermocycler using the following cycling conditions: 95 °C for 5 min, ramp down to 25 °C at 5 °C/min.

#### 3.2.2 pX330 Linearization

1. On ice, prepare a 50 μL reaction containing 100 ng circular pX330 vector, 5 μL restriction enzyme buffer (10 ×), 4 μL BbsI (5000 U/mL), and Milli-Q water to a final volume of 50 μL.
2. Incubate for 2 h at 37 °C.

#### 3.2.3 Ligation of Annealed Oligos into pX330

1. Dilute annealed oligos (from Subheading 3.2.1)1:200 in Milli- Q water.
2. Prepare a 10 μL reaction mix containing 50 ng of linear pX330 (from Subheading 3.2.2), 1 μL diluted oligos, 1 μL 10× T4 ligase buffer, 1 μL T4 ligase enzyme, and Milli-Q water to a final volume of 10 μL.
3. Incubate for at least 1 h at room temperature, or overnight at 4 °C.

#### 3.2.4 Transformation of Competent Cells with the Ligation Mix and Isolation of Plasmid DNA

1. Thaw *E. coli* JM109 competent cells on ice until just thawed.
2. Add 5 μL of the ligation reaction (from Subheading 3.2.3) to the tube of competent cells. Mix by flicking the tube 4–5 times (do *not* mix by pipetting) and incubate cells for 30 min on ice.
3. Heat shock the cells for 45 s in a water bath at exactly 42 °C; do not shake. Immediately return the tube to ice for 2 min.
4. Add 450 μL room-temperature SOC medium to the tube and incubate for 1 h at 37 °C with shaking.
5. Plate 100 μL of the transformation mix onto an LB agar Amp plate.
6. Incubate plate overnight at 37 °C.
7. The next day, inspect the plate for colony growth. Pick two or three colonies to check for correct insertion of the sgRNA. Inoculate a single colony into a 5 mL culture of LB Amp medium. Incubate cultures overnight at 37 °C with shaking.
8. Isolate plasmid DNA from the cultures using a QIAprep Spin Miniprep kit, according to the manufacturer’s instructions.
9. Verify the sequence of each plasmid by Sanger sequencing using the pX330 sequencing primer. We will call this plasmid pX330-sgRNA.

### 3.3 Transfection of HEK293 or IMCD3 Cells

A different transfection reagent is used depending on the cell line (*see* **Note 7**). Choose Subheading 3.3.2 for transfection of HEK293 cells and Subheading 3.3.3 for transfection of IMCD3 cells. Regardless of transfection reagent, three transfection conditions will be used: (1) a co-transfection with pX330-sgRNA and pAcGFP1-C1 (“CRISPR” transfection); (2) a transfection with pAcGFP1-C1 (“GFP-only” transfection); (3) a mock transfection without plasmid DNA (“non-transfected” condition). The latter two conditions act as positive and negative controls of transfection, respectively.

#### 3.3.1 Seeding of Cells for Transfection

1. The day before transfection, seed cells into three wells of a 6-well plate (*see* **Note 8**). Plate 1 × 10^5^ to 3 × 10^5^ cells/well in 2 mL/well complete medium. Leave overnight to achieve a desired confluence of 50–80%, appropriate for transfection.

#### 3.3.2 Transfection of HEK293 Cells

1. Label three sterile microcentrifuge tubes, one for each transfection condition.
2. In each tube, mix 3 μL FuGENE with 97 μL Opti-MEM reduced-serum media and incubate for 5 min at room temperature.
3. Add the appropriate plasmid DNA to each tube and mix. For the CRISPR transfection, add 500 ng pX330-sgRNA and 500 ng pAcGFP1-C1. For the GFP-only transfection, add 1 μg pAcGFP1-C1. No plasmid DNA is added for the non-transfected condition. Incubate for 15 min at room temperature.
4. To each well of plated cells, add 100 μL of transfection mixture in a dropwise manner.
5. Return cells to the incubator for 48 h, prior to cell sorting (*see* **Note 9**).

#### 3.3.3 Transfection of IMCD3 Cells

1. Label six sterile microcentrifuge tubes. For each transfection condition, there will be one tube for Lipofectamine and one tube for plasmid DNA.
2. In each of the three tubes, mix 8.5 μL Lipofectamine 2000 transfection reagent with 150 μL Opti-MEM and incubate for 5 min at room temperature.
3. In the remaining three tubes, mix 150 μL Opti-MEM with the appropriate plasmid DNA. For the CRISPR transfection, add 1.25 μg pX330-sgRNA (obtained in step 3.2.7) and 1.25 μg pAcGFP1-C1. For the GFP-only transfection, add 2.5 μg pAcGFP1-C1. No plasmid DNA is added for the non-transfected condition.
4. Add each plasmid mixture to a separate tube of Lipofectamine and mix well. Incubate for 30 min at room temperature.
5. To each well of plated cells, add 300 μL of transfection mixture in a dropwise manner.
6. Return the cells to the incubator for 48 h, prior to cell sorting (*see* **Note 9**).

### 3.4 Group Sort by Fluorescence- Activated Cell Sorting (FACS)

1. Label a 15 mL centrifuge tube for use as a CRISPR sample collection tube. Add 500 μL fresh medium to the tube, put it on ice, and set aside.
2. Wash the CRISPR and non-transfected wells of cells twice each with 1 mL/well sterile DPBS (*see* **Note 10**).
3. Add 1 mL/well trypsin-EDTA and incubate at 37 °C until cells dissociate.
4. Add 2 mL/well complete media and transfer each 3 mL cell suspension to a 15 mL centrifuge tube.
5. Pellet cells by centrifugation at 200 × *g* for 5 min.
6. Discard the supernatant and resuspend cells in 300 μL Opti- MEM reduced-serum media (*see* **Note 11**).
7. Pass cells through a 70 μm cell strainer to remove clumps (*see* **Note 12**).
8. Put the tubes directly on ice.
9. Proceed directly to FACS (*see* **Note 13**). Use non-transfected cells to discern single cells from cell clumps when setting up the cell sorter. Isolate GFP-positive cells from the CRISPR sample and collect these in the collection tube (prepared in Subheading 3.4, **step 1**). Run the entirety of the sample or collect at least 5 × 10^5^ cells per sample (*see* **Note 14**). Keep collected cells on ice.
10. In a sterile safety cabinet, take one-half of the GFP-positive CRISPR sample and seed them in a 25 cm^2^ tissue culture- treated flask containing 5 mL complete medium. Return the cells to the incubator and continue to grow them for 2 weeks, passaging as and when necessary (*see* **Note 15**).
11. Separately pellet the remaining GFP-positive CRISPR cells and the non-transfected cells for gDNA extraction, by centrifugation at 300 × *g* for 5 min. Discard the supernatant. Proceed directly to the next step, or store the cell pellets at –20 °C.

### 3.5 T7 Endonuclease I (T7EI) Assay

**Fig. 3:**
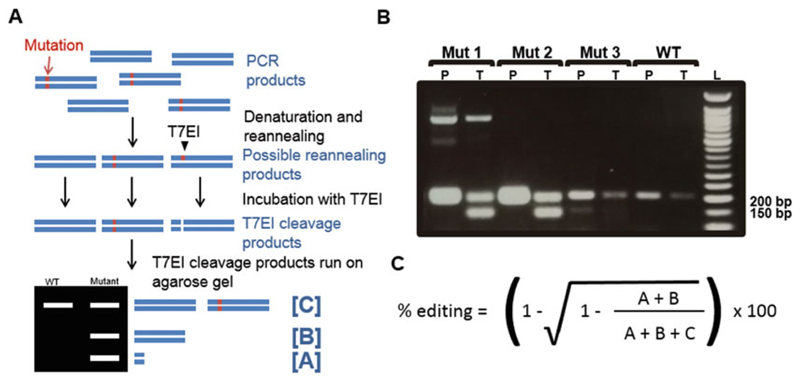
T7EI assay. (**a**) Overview of the T7EI assay: The T7EI assay is used to detect indels at the genomic target site. The target site is first PCR-amplified and the PCR products denatured and reannealed. If a mutation is present, reannealing products include WT homoduplex DNA, mutant homoduplex DNA, and WT/DNA heteroduplex DNA. The T7EI enzyme identifies and cleaves DNA mismatches. Incubation of reannealing products with T7EI therefore results in cleavage of heteroduplex DNA, whereas homoduplex DNA is left intact. When run on an agarose gel, T7EI cleavage products can be detected. (**b**) Example PCR and T7EI assay results. PCR (P) and T7EI assay (T) products of three group-sorted CRISPR populations (Mut 1,2,3) and a wild-type (WT) were run alongside a DNA ladder (L) in an agarose gel. The expected PCR amplicon size was 204 bp. In the WT sample, the 204 bp amplicon was not cleaved by the T7EI. Mutant 1 was a heterogeneous population, revealed by the presence of more than two PCR amplicons. Mutant 2 had small indels at the target site, revealed by a single PCR amplicon that is cleaved by the T7EI. Mutant 3 possessed an allele with a large deletion, revealed by the presence of an additional, smaller PCR amplicon. (**c**) Calculation of targeting efficiency. Assuming that all indels are not identical, the equation approximates the targeting efficiency, where A, B, and C refer to band intensities labeled in (A)

1. Extract genomic DNA (gDNA) with the DNeasy Blood and Tissue kit, following the manufacturer’s instructions.
2. Use the extracted gDNA as template to perform a 50 μL PCR reaction using the genotyping primers (designed in Subheading 3.1, **step 10**) and Pwo Master (*see* **Note 16**).
3. Run 25 μL of the PCR in an agarose gel (adjusting the agarose percentage depending on the expected size of the PCR product) against a 1 kb DNA ladder to check whether there is a single product (*see* **Note 17**).
4. In a 0.2 mL PCR tube, prepare a 19 μL reaction containing 10 μL PCR reaction, 2 μL 10 × NEBuffer 2, and 7 μL Milli-Q H2O.
5. Melt the PCR products in a thermocycler using the following conditions: 95 °C for 5 min, ramp down to 85 °C at –2 °C/s, ramp down to 25 °C at –0.1 °C/s, and hold at 4 °C.
6. Add 1 μL T7 endonuclease I to the melted PCR products and incubate at 37 °C for 1 h.
7. Run the T7 cleavage products in an agarose gel and look for cleavage of the PCR product (*see* **Notes 17** and **Note 18**; Fig. 3).

### 3.6 Single-Cell Sorting of CRISPR Cells to Establish Clonal Cell Lines

#### 3.6.1 Preparation of Conditioned Media

1. In a 50 mL centrifuge tube, make up 16 mL of conditioned medium per 96-well plate (*see* **Note 19**), by mixing 8 mL fresh medium with 8 mL medium removed from a 75 cm^2^ flask of cells at ~70% confluence (*see* **Note 20**).
2. Sterile-filter the conditioned medium with a 0.2 μm filter (*see* **Note 21**).
3. Fill a 96-well plate with 150 μL/well. Place the 96-well plate in the 37 °C incubator.

#### 3.6.2 Preparation of CRISPR Cells for FACS

1. Wash cells in 25 cm^2^ flask twice with 5 mL sterile DPBS.
2. Add 1 mL trypsin-EDTA and incubate at 37 °C until cells dissociate.
3. Add 4 mL complete media and transfer cell suspension to a 15 mL centrifuge tube.
4. Pellet cells by centrifugation at 200 × *g* for 5 min.
5. Discard the supernatant and resuspend cells in 300 μL Opti- MEM reduced-serum media (*see* **Note 11**).
6. Pass cells through a 70 μm cell strainer to remove clumps (*see* **Note 12**).
7. Put the tubes directly on ice.
8. Proceed directly to FACS with the cell suspension and the 96-well plate containing conditioned media. Sort a single cell into each well of the 96-well plate (*see* **Note 22**).
9. Spin the 96-well plate at 200 × *g* for 2 min and immediately return them to the incubator (*see* **Note 23**).

#### 3.6.3 Expansion of Cell Colonies

1. Incubate the cells for 2 weeks, to allow colonies to establish (*see* **Note 24**).
2. After 2 weeks, check individual wells under the microscope to identify those that contain a cell colony. Monitor cell confluence in these wells over the ensuing days.
3. When a well is confluent (*see* **Note 25**), wash the cells twice in 200 μL sterile DPBS. Then dissociate cells in 30 μL trypsin- EDTA and transfer them directly into 1 mL medium in a well of a 12-well plate (*see* **Note 26**). Return the cells to the incubator and monitor their growth over the following days.
4. When a well of a 12-well plate is confluent, wash the cells twice with 1 mL sterile DPBS. Then dissociate cells in 150 μL trypsin-EDTA and transfer directly to a 25 cm^2^ flask containing 5 mL medium. Return the cells to the incubator and monitor their growth over the following days.
5. When a 25 cm^2^ flask is confluent, passage the cells into two new 25 cm^2^ flasks. Return these flasks to the incubator.
6. When the two 25 cm^2^ flasks have reached ~70% confluence, freeze down and store the cells from one of the flasks.
7. With the remaining flask, wash cells twice with 5 mL DPBS, and then dissociate in 1 mL trypsin-EDTA. Add 4 mL complete media and transfer the 5 mL cell suspension to a 15 mL centrifuge tube. Pellet the cells for 5 min at 300 × *g* and discard the supernatant. Proceed directly to the next step, or store the cell pellet at –20 ° C.

### 3.7 Genotyping of Clones

This method describes the genotyping of a single-cell colony.

**Fig. 4:**
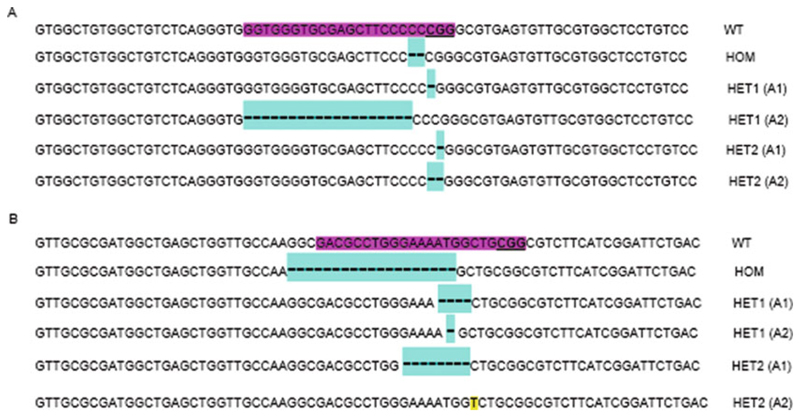
Different mutations found in each cell line after sequencing. Homozygotes and compound heterozygotes have been found in both HEK 293 and IMCD3 cells. (**a**) Mutations found in single clones in HEK 293 cells after Sanger sequencing. (**b**) Mutations found in single clones in IMCD3 cells after Sanger sequencing. The sgRNA is highlighted in purple and the PAM sequence is underlined in WT samples. Note that the cyan color shows deleted bases and yellow indicates inserted bases

1. Extract genomic (g) DNA with the DNeasy Blood and Tissue kit, following the manufacturer’s instructions.
2. Use the extracted gDNA as template to perform a 50 μL PCR reaction with the genotyping primers (designed in Subheading 3.1, **step 10**).
3. Run the 50 μL PCR in an agarose gel against a 1 kb DNA ladder. If there are multiple amplicons present, ensure that they are well resolved (*see* **Notes 27** and **28**).
4. Gel-purify the PCR products.
5. Sanger sequence the extracted amplicon(s) to identify the presence and nature of mutations generated by CRISPR (*see* **Note 29**; Fig. 4).

## 4 Notes

**Fig. 5:**
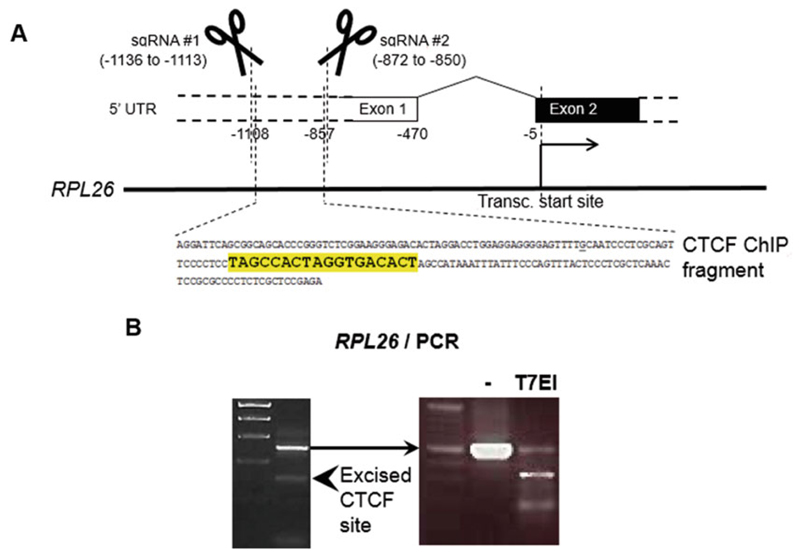
Example of using pairs of sgRNAs for efficient excision of a DNA fragment. (**a**) In this example, a predicted CTCF-binding site (highlighted in yellow) within a CTCF ChIP fragment was excised using two sgRNAs located 239 nt apart. (**b**) PCR products were used to amplify the region surrounding these sgRNA- binding sites leading to a significant level of successful targeting, with excision of the desired sequence (arrowhead, left). The undigested fragment (indicated by the arrow) contained a high level of likely indels, as demonstrated by T7EI digestion of this product (right)

1. The given methods describe the use of the NHEJ pathway to knock out a gene by generating random indels. We have adapted these methods to knock in a specific point mutation in IMCD3 cells using the HDR pathway, by integrating methods from the literature [9, 10, 16]. The basic protocol presented in this chapter can be followed, making the following three changes:
  (a) In Subheading 3.1, the sgRNA must be designed to target the Cas9 to cut as close to the point at which the single base change is to be introduced, ideally within 10 bp. The necessity of a PAM site being proximal to the desired mutation site is a constraint of the HDR method.
  (b) A repair template (single-stranded oligodeoxynucleotide, ssODN) is required. This must contain the desired base change flanked by 60 nt homology arms, as well as a silent PAM mutation. Mutation of the PAM site prevents further Cas9 cleavage after mutation generation. It is important that the PAM mutation is silent to avoid introduction of an unwanted missense mutation. The ssODN should be ordered as an oligonucleotide and resuspended in Milli-Q water to a concentration of 10 μM, prior to proceeding to Subheading 3.3.
  (c) The CRISPR transfection of IMCD3 cells (in Subheading 3.3) must accommodate the ssODN. For the CRISPR transfection, add 1.2 μg pX330-sgRNA (obtained in step 3.2.7), 1.2 μg pAcGFP1-C1, and 1 μl ssODN (10 μM).
2. We have found that HEK293 cells can be transfected with high efficiency using the FuGENE 6 reagent (Promega). In IMCD3 cells, higher transfection efficiency was achieved with the Lipo-fectamine 2000 transfection reagent (Thermo Fisher) than with FuGENE.
3. If you are not interested in editing a specific locus, the chance of gene knockout will be improved by choosing a sgRNA target site that is in an early exon of the gene. However, it is also worth assessing whether internal translation initiation codons are present within the gene, as these could generate transcripts not affected by a frameshift mutation.
4. Several online sgRNA design tools are now available. We have successfully used CRISPOR (crispor.tefor.net), Chopchop (chopchop.cbu.uib.no), and Benchling (benchling.com) to design sgRNAs. Chopchop does not require the genomic region of interest to be pasted into the search box; instead, the gene of interest can be searched for within the appropriate genome, and all possible sgRNA options spanning the entire gene are presented.
5. When choosing a sgRNA it is important to consider its score (out of 100). A high score indicates a faithful sgRNA. A low score indicates a sgRNA with predicted off-target activity. For each sgRNA, off-target loci (including those with mismatches) are predicted, which are listed when the sgRNA is selected. The number of off-target sites within genes is also listed. Select a high-scoring sgRNA that does not have any predicted off-target sites in genes.
6. Design primers to generate an amplicon of 200–500 bp. The expected Cas9 cut site (~3 bp upstream of the “NGG” PAM) should not lie centrally within the amplicon, or else two products of roughly equal size will be produced by the T7EI assay and it will not be possible to resolve these by gel electrophoresis. It is recommended that the target genomic region, including the primer-binding sites, is Sanger sequenced within the cell line of choice. Primers should be designed to generate an amplicon that does not contain monoallelic mutations; these will give false positives in the T7EI assay and may bias primer binding (if they fall within primer-binding sites).
7. In a variation of the method presented here, two sgRNAs can be used in combination to excise a genomic region. In this case, two sgRNAs should be designed in Subheading 3.1. Each can be independently cloned into a pX330 vector, to generate two separate pX330-sgRNA plasmids, one for each sgRNA. Trans-fection conditions can be adapted (in Subheading 3.3), to accommodate transfection of two pX330-sgRNA plasmids with the pAcGFP1-C1 plasmid. Larger deletions can be detected by running PCR products on an agarose gel (Fig. 5).
8. The procedure can be adjusted for other culture vessel sizes; scale reagents as necessary.
9. Confirm GFP expression in CRISPR and GFP-only cells by fluorescence microscopy 18–24 h post-transfection. The GFP-only cells are used purely as a control for transfection, and can be discarded prior to the group sort.
10. When preparing cells for FACS, take extra care not to cross-contaminate the different samples. The GFP-only cells do not need to be prepared for FACS (*see* **Note 9**).
11. It is important to use serum-free medium so as not to clog the FACS apparatus with sera.
12. Cell straining is optional, although it is advised, particularly for IMCD3 cells. This will reduce cell clumps.
13. We use a MoFlo XDP (Beckman Coulter) equipped with three air-cooled lasers: a 150 mW 488 nm argon laser, a 100 mW 643 nm red laser, and a 100 mW 355 nm UV laser. A 100 nm nozzle tip is used with 30 PSI sheath pressure. eGFP signal is collected in FL1 channel through a 530/40 band-pass filter and FL3 channel with a 613/20 band-pass filter is used to separate true GFP-positive cells from autofluorescence background. A light scatter gate drawn in the FSC versus SSC plot is used to exclude debris and clumps and include viable cells. Cells in this gate are displayed in a SSC versus SSC-W to further target single cells. Single and viable cells are then analyzed in a FL3 versus FL1 plot and a final gate is drawn on green cells for cell sorting.
14. It is possible to complete the protocol if less than 5 × 10^5^ cells are collected. In this case, prioritize cells for seeding into a culture vessel (Subheading 3.4, **step 10**); use a vessel of a size appropriate to the number of cells being seeded. If too few cells remain for gDNA extraction (Subheading 3.4, **step 11**, to Subheading 3.5, **step 1**), split some of the reseeded cells for gDNA extraction once they have begun to proliferate.
15. We have observed that single-cell sorting cells 2–3 days post-transfection tends to give rise to heterogeneous populations of cells, rather than clonal colonies (they possess more than two alleles when genotyped). We hypothesize that this is due to continued expression of the sgRNA and Cas9 in daughter cells, after division of the single-cell sorted cell. We passage cells for 2 weeks, to await loss of pX330 plasmid from the cells.
16. Optimize PCR conditions according to your needs. Pwo poly-merase may be substituted for another polymerase of your choice. Ensure that the polymerase is of high fidelity and/or possesses proofreading ability (3′ to 5′ exonuclease activity); errors during PCR amplification will give false positives in the T7EI assay.
17. Use non-transfected cells as a control; these should behave as WT to give a single PCR product which is not cut by the T7EI assay. Multiple PCR products may be resolved from the CRISPR sample if it contains alleles with large indels. If there is a significant size difference between these PCR products, the T7EI assay may produce more than three bands on the gel.
18. sgRNA efficiency can be determined from the intensity of the products resulting from T7EI assay of the CRISPR sample: compare the intensity of the uncut product to that of the cleavage products; the brighter the cleavage products (relative to the uncut product), the more efficient the sgRNA. If T7EI assay of the CRISPR sample does not result in cleavage products, sgRNA activity may be inefficient, and design of a new sgRNA should be considered.
19. Use as many 96-well plates as needed. We typically use 2–3 plates to maximize the number of colonies obtained.
20. It is necessary to passage some cells (of the same line) 24–48 h before preparing transfected cells for FACS, in order to formulate conditioned medium. Conditioned medium is used to improve the likelihood of cell survival after the single-cell sort. It comprises fresh medium (containing necessary nutrients) and medium taken from cells at ~70% confluence. The latter contains signaling components secreted by healthy cells in exponential growth phase, to encourage the sorted single cells to grow.
21. Conditioned medium must be sterile-filtered to remove contaminating cells, which may derive from media taken from cells at ~70% confluence. Contaminating cells will obstruct the establishment of clonal cell lines.
22. The majority of cells will no longer be GFP positive. Isolate any single cells, regardless of GFP expression.
23. Centrifugation of the 96-well plate improves chances of cell adhesion, by moving cells to the bottom of the plate. It is important to get the single-cell sorted cells into the incubator as quickly as possible after FACS, to maximize viability.
24. Changing medium is not necessary; this risks loss of cells from the wells.
25. It may be necessary to passage the cells before the entire well is confluent, if cells become dense but do not spread across the well, and media become orange/yellow in color.
26. Confirm cell detachment under the microscope prior to trans-ferring cells to the new culture vessel. Transfer the full volume of cell suspension to the 12-well plate without pelleting cells. Then check the (empty) well of the 96-well plate under the microscope again, to ensure that all cells have been transferred. Loss of cells during passage will result in reduced cell density in the 12-well plate, which may decrease cell viability.
27. Presence of more than two PCR products indicates that the colony is a clone with aneuploidy, or that it is not clonal (it is a heterogeneous population).
28. It is possible to perform the T7EI assay to genotype clones. However, note that T7EI will not cleave PCR products derived from homozygous mutants. T7EI will only give a positive result (cleavage) in PCR products derived from heterozygotes or compound heterozygotes.
29. If Sanger sequencing produces two overlapping traces, the clone is a heterozygote or a compound heterozygote. Cloning of PCR products in pGEM-T Easy vector systems (Promega) can be used to isolate allelic sequences. If more than two overlapping traces are seen, the colony is a clone with aneuploidy, or it is not clonal (it is a heterogeneous population).
